# Hydrogen‐Bonding Crosslinking MXene to Highly Robust and Ultralight Aerogels for Strengthening Lithium Metal Anode

**DOI:** 10.1002/smsc.202100021

**Published:** 2021-07-16

**Authors:** Xiangyu Meng, Yufeng Sun, Mengzhou Yu, Zhiyu Wang, Jieshan Qiu

**Affiliations:** ^1^ State Key Lab of Fine Chemicals Liaoning Key Lab for Energy Materials and Chemical Engineering Dalian University of Technology Dalian 116024 China; ^2^ State Key Laboratory of Space Power-Sources Technology Shanghai Institute of Space Power-Sources Shanghai 200245 China; ^3^ College of Chemical Engineering Beijing University of Chemical Technology Beijing 100029 China

**Keywords:** highly robust Li metal anode, Li metal anode, MXene aerogels, rechargeable batteries, ultralight aerogels

## Abstract

Li metal batteries offer the ultimate choice of high‐energy power source, but suffer the performance decay and safety risk originated from notorious dendrite problem and infinite volume change of Li metal anode. Herein, it is reported to strengthen the Li metal anode by ultralight but highly robust MXene aerogels (ULRMA) with build‐in strain‐resistant and molecular‐level lithiophilic properties. A hydrogen‐bonding crosslinking strategy is developed for rapidly assembling 2D MXene to ULRMA at ambient conditions with less sacrifice of intrinsic properties of MXene. The ULRMA with an ultralow density below 10 mg cm^−3^ are favorable to maximize the merit of Li metal in gravimetric energy density while offering exceptional stability of porous frameworks against mechanical strain of long‐term Li plating/stripping. The lithiophilic architecture with high conductivity and hierarchical porosity further largely reduces the potential polarization and guides the pattern of Li deposition. Uptaking Li metal into ULRMA leads to a stable Li metal anode with an ultralong lifetime of 1600 h with a high‐rate response up to 20 mA cm^−2^ and high coulombic efficiency. It yields a highly robust Li metal anode with high effectiveness in engineering stable Li‐ion and Li–S batteries even paring with commercial LiFePO_4_ or sulfur cathode without nanostructuring.

## Graphical abstract

A facile hydrogen‐bonding crosslinking strategy is developed for fast fabrication of ultralight but highly robust MXene aerogels (ULRMA). They allow for maximizing the gravimetric energy merit of Li metal while effectively strengthening Li metal anode. Uptaking Li metal into robust lithiophilic ULRMA yields stable Li metal anode for significantly enhancing the performance of Li‐ion and Li–S full cells.

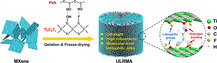

## 1 Introduction

Li metal has been recognized as the ultimate choice of anode material for next‐generation rechargeable batteries due to its ultrahigh theoretical capacity (3860 mAh g^−1^), low density (0.534 g cm^−3^), and very low redox potential (−3.04 V vs standard hydrogen electrode) among all solids.^[^ ^1^ ^]^ They could deliver over tenfold higher capacities than that of commercial carbon‐based anodes, offering the ultimate opportunities for boosting the battery energy density far beyond the existing Li‐ion batteries.^[^ ^2^ ^]^ However, the operating performance of Li metal anode is severely harmed by repeated and uncontrolled Li plating/stripping on the coarse electrode–electrolyte interface. This effect not only induces huge and infinite volume change of Li metal anode to damage it, but also leads to nonequilibrium growth of Li metal to sharp dendrites, causing the penetration of separator to short‐circuit the cells. Upon Li stripping, uneven dissolution of Li dendrites further generates the volume of “dead Li metal” that is electrically isolated from the electrode interface, thereby reducing the cell reversibility. In addition, the Li metal with a high Fermi energy level shows extremely high reactivity with nearly all electrolytes.^[^ ^3^ ^]^ Such side reactions inevitably cause irreversible Li loss and fast depilation of the electrolyte by forming a thick solid electrolyte interphase (SEI) with high ionic/charge transport resistance. Macroscopically, all these problems manifest as fast cell failure and poor reliability of Li metal batteries, which fundamentally limit their practical potential for a long shot.^[^ ^4^ ^]^

Various strategies have been proposed to strengthen the Li metal anodes in terms of artificial interface engineering, modifying electrolyte composition and/or phase state, tailoring lithiophilic architecture, and regulating the discharge–charge protocol.^[^ ^5^ ^]^ On this basis, the Li hosts with 3D porous architecture were further utilized for achieving even Li plating/stripping by homogenizing the Li^+^ mass transfer and localized electric field.^[^ ^6^ ^]^ Large surface area and sufficient free space of such anode configuration bring particular benefits in minimizing the huge volume change of the anode with heavy Li metal loading.^[^ ^7^ ^]^ These merits allow one to suppress the Li dendrite growth at high current density with less sacrifice of Li metal loading and anode kinetics. Porous metal (e.g., Cu and Ni) foams were commonly used to provide ample space for hosting Li metal effectively.^[^ ^8^ ^]^ However, their poor lithiophilic nature makes the Li deposition difficult with high nucleation overpotential and low energy efficiency. Modifying the metal foams with lithiophilic nanoparticles may guide the Li nucleation via chemical interaction or alloying reactions.^[^ ^9^ ^]^ Nevertheless, the large grain size of such Li‐anchoring sites restricts the uniformity of Li plating/stripping on the host materials. More seriously, the metal foams with far higher or comparable densities (e.g., 3.3 g cm^−3^ for Cu foam and 0.45 g cm^−3^ for Ni foam, Sigma‐Aldrich) to Li metal (0.534 g cm^−2^) largely offset the merits of Li metal batteries in gravimetric specific energy.^[^ ^10^ ^]^ Porous carbon or polymer host with lightweight and high flexibility offers a promising material platform for addressing these problems.^[^ ^11^ ^]^ Nevertheless, the lithiophilic ability of carbon hosts is limited by their intrinsically nonpolar surface with weak interaction with Li metal. This fundamental difficulty could be addressed by chemical functionalization, defect engineering, or structural doping of carbon materials. It, however, adversely damages the intrinsic electronic structures of carbon host to limit the potential in engineering high‐performance Li metal anodes,^[^ ^10^ ^]^ whereas the poorly conductive or insulating polymers are even inferior for smooth Li deposition, making the practical use more difficult.^[^ ^12^ ^]^

In recent years, the MXene emerged as a new class of 2D functional materials collecting various attractive properties, such as high mechanical robustness, wide chemical variety, and superb conductivity associated with a high electron density of states nearby Fermi level and high carrier density.^[^ ^13^ ^]^ In general, they share a formula of M_*n*+1_X_*n*_T_*x*_ (*n* = 1–3), where M is early transition metals (e.g., Ti, Mo, Nb, Ta, and V), X is the C and/or N elements, and T stands for the chemical groups such as —OH, —O, and —F on the surface.^[^ ^14^ ^]^ Compared with lithiophilic nanograins, these electron‐donating groups offer a natural abundance of molecular‐level lithiophilic sites on MXene for guiding uniform nucleation and deposition of Li metal more efficiently.^[^ ^15^ ^]^ Recent work also suggested that some groups such as —F is favorable to in situ create a LiF‐rich SEI with high Li^+^ conductivity for improving the high‐rate performance of Li metal anode.^[^ ^16^ ^]^ Nevertheless, the MXene tends to aggregate via intersheet van der Waals force and/or hydrogen bonding, especially under huge strain during Li metal plating/stripping.^[^ ^17^ ^]^ This drawback severely reduces the availability of lithiophilic interface and interspacing room for hosting Li metal, limiting the full exploitation of the potential of MXene in strengthening Li metal anodes. Engineering the aerogels or polymer‐foam supported 3D MXene architectures has been considered to enlarge the lithiophilic interface for hosting more Li metal.^[^ ^18^ ^]^ However, a major problem of usual 3D MXene assemblies is the brittle structure with quite poor mechanical strength. As a result, they hardly survive from the heavy and infinite strain caused by repeated Li deposition/stripping, losing Li storage room and lithiophilic interface to fundamentally restrict the long‐term reversibility and stability of Li metal anodes.

In this work, we report a facile hydrogen‐bonding crosslinking strategy for fast fabrication of ultralight but highly robust MXene aerogels (ULRMA) at ambient conditions. The strategy endows the MXene aerogels (MAs) with an ultralow density but exceptional mechanical strength with less sacrifice of intrinsic properties of MXene. The ULRMA with build‐in strain‐resistant properties, molecular‐level lithiophilic sites, high conductivity, and hierarchical porous architecture can serve as an ideal host for uptaking Li metal. They allow for maximizing the gravimetric energy of Li metal while effectively strengthening the Li metal anode, enabling excellent long‐term stability and reversibility without dendrite hazards. It significantly improves the performance of Li‐ion and Li–S cells by paring with the commercial cathodes, implying the great potential in building long‐life and high‐energy‐density batteries.

## 2 Results and Discussion

**Figure 1:**
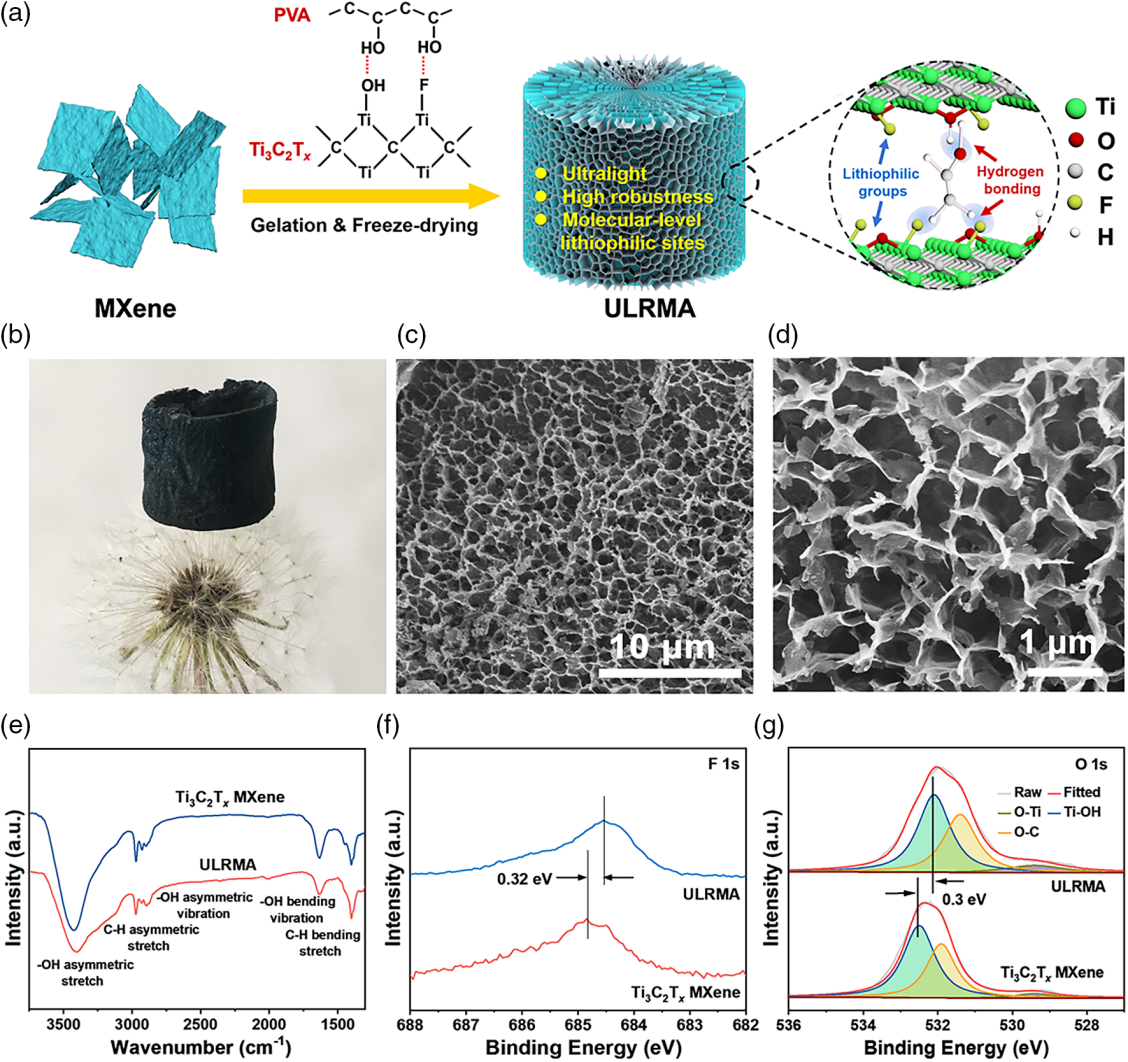
a) Schematic illustration of the fabrication strategy of ULRMA. b) Optical images of ULRMA standing on a dandelion flower. c,d) SEM images of ULRMA with hierarchically porous 3D architecture. e) Fourier transform infrared (FT‐IR), f) F 1s, and g) O 1s XPS spectra of ULRMA and pristine Ti_3_C_2_T_*x*_ MXene.

The synthetic strategy of ULRMA is schematically shown in **Figure** **1**a. The high‐quality Ti_3_C_2_T_*x*_ MXene with a lateral size of hundreds of nanometers is first fabricated by selectively etching the layered Ti_3_AlC_2_ MAX phase in LiF/HCl solution, followed by ultrasonic exfoliation to thin nanosheets (Figure S1, Supporting Information).^[^ ^19^ ^]^ A hydrogel can be rapidly prepared by simply aging the aqueous colloids of Ti_3_C_2_T_*x*_ MXene in the presence of polyvinyl alcohol (PVA) for 1 h at room temperature. In this process, the PVA molecular chains with substantial hydroxyl groups and strong gelation capability act as chemical crosslinkers. They induce rapid self‐assembly of 2D MXene to 3D frameworks via hydrogen bonding with the —OH and —F groups on the surface of MXene. The water in such hydrogels is removed by freeze‐drying to yield ULRMA consisting of 3D interconnected MXene nanosheets with interconnected macropores ranging from tens to hundreds of micrometers (Figure 1b–d). The ULRMA share a similar X‐ray diffraction (XRD) pattern with pristine MXene. The fingerprint (002) peaks shift to a lower angle due to the PVA intercalation into the layered space of MXene, which effectively suppressed intersheet restacking of MXene (Figure S2, Supporting Information).^[^ ^20^ ^]^ The ULRMA have a hierarchical meso‐macroporous structure with a specific surface of 76.91 m^2^ g^−1^, which is over three times as high as that of pristine MXene (25.23 m^2^ g^−1^) (Figure S3, Supporting Information). Such a highly porous architecture is highly favorable to the fast accessibility of a large lithiophilic interface to Li^+^ flux and Li deposition while offering ample space for uptaking a high volume of Li metal and accommodating their huge volume fluctuation upon cycling.

The hydrogen bonding between the hydroxyl groups of PVA and surface terminations of Ti_3_C_2_T_*x*_ MXene is confirmed by the broadening and shift of —OH absorption bands toward the lower wavenumber after MXene gelation with PVA (Figure 1e).^[^ ^21^ ^]^ The X‐ray photoelectron spectrometer (XPS) survey scan indicates the coexistence of Ti, C, O, and F elements in ULRMA (Figure S4a, Supporting Information). For pristine MXene, the F 1s XPS spectrum features with a peak at 684.9 eV associated with C—Ti—F bond (Figure 1f).^[^ ^22^ ^]^ This peak is negatively shifted to lower binding energy by 0.32 eV in ULRMA via the —F…H—O interaction between the —F on MXene and —OH groups of PVA. The —O…H—O interaction between —OH on MXene and —OH of PVA is verified by the negative shift of the peak from Ti—OH bonds (0.3 eV) relative to pristine MXene in the O 1s XPS spectrum (Figure 1g). The Ti 2p XPS spectrum of ULRMA can be deconvoluted into three pairs of 2p3/2/2p1/2 doublets for Ti—C (454.9/460.6 eV), Ti^2+^ (456/461.7 eV), and Ti^3+^ (457.2/462.9 eV) (Figure S4b, Supporting Information). These peaks show a negligible shift with respect to pristine Ti_3_C_2_T_*x*_ MXene, ruling out the chemical interaction between Ti atoms in MXene and PVA.^[^ ^23^ ^]^ As a result, the electronic structure and electrical properties of Ti_3_C_2_ backbones could be well reserved. The Raman spectra of ULRMA show the modes at ≈201 and 729 cm^−1^ for A1g symmetry out‐of‐plane vibrations of Ti and C atoms, and the ones at 279, 380, and 573 cm^−1^ for the *E*_g_ vibrations of in‐plane Ti, C, and surface functional group atoms in MXene (Figure S5, Supporting Information).^[^ ^24^ ^]^ It shares a similar feature with pristine Ti_3_C_2_T_*x*_ MXene, suggesting that the hydrogen‐bonding crosslinkage negligibly affects the intrinsic electronic properties of MXene in ULRMA. The electrical conductivity of ULRMA is ≈60 S cm^−1^, which is favorable to facilitate the charge transfer and reduce the Li^+^ concentration polarization nearby electrode interface.

**Figure 2:**
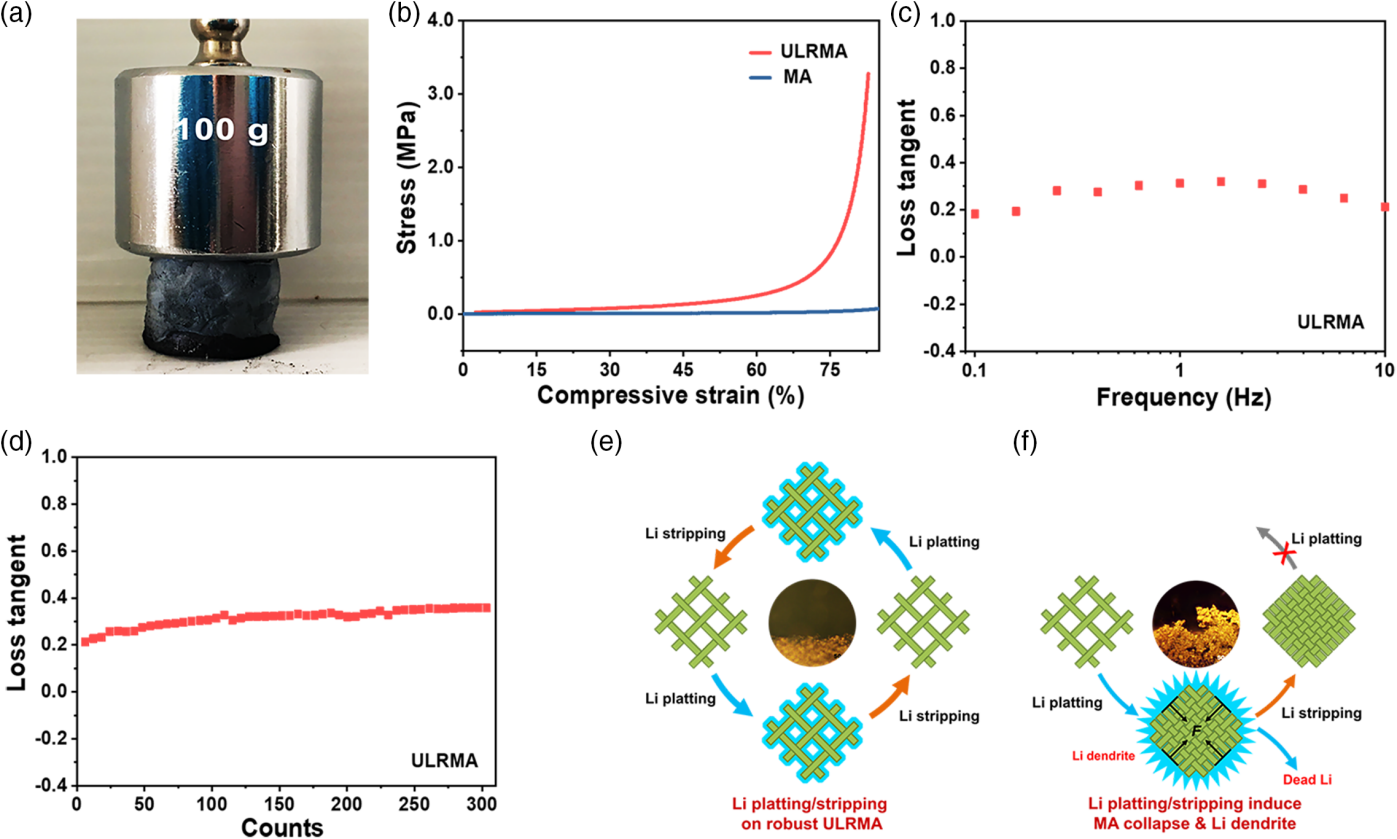
a) Optical image of a ULRMA pressed under a weight of 100 g. b) Stress–compressive strain curves of ULRMA and MA. A curve of loss tangent versus c) frequency and d) time at 1.0 Hz with an oscillatory strain of 0.5% for ULRMA. Schematic illustration of Li plating/stripping on e) ULRMA and f) MA.

Firmly locking of MXene nanosheets by PVA molecular chains endows the ULRMA with significant improvement in mechanical robustness as compared with physically interconnected MAs (Figure S6, Supporting Information). The ULRMA could withstand a 900‐fold higher weight loading without apparent deformation and fracture (**Figure** **2**a). It gives rise to a high Young's modulus of 260 kPa, which far exceeds the MA by over 16‐fold (16 kPa) (Figure 2b). Compressing the volume of ULRMA by 80% requires a high pressure of 1.72 MPa (Figure 2b). Moreover, the ULRMA also exhibit good mechanical resilience with a low loss tangent (≈0.3) at a frequency range of 0.1–10 Hz (Figure 2c). This feature can be steadily maintained for 300 cycles of the tests, implying good recoverability of the structure against repeated strain (Figure 2d). High mechanical robustness with structure recoverability endows the ULRMA with excellent build‐in strain‐resistant properties. It is undoubtedly important to prevent the porous architecture of ULRMA from collapse and allows it to deal with the huge and repeated mechanical strain caused by infinite Li plating/stripping with ease on substantial lithiophilic interface (Figure 2e,f). This merit also benefits to maintain high accessibility of electrode interface to fast Li^+^ flux and large current, thereby enhancing the performance of Li metal anode upon rapid plating/stripping at high rates. On the contrary, the physically interconnected MA fast collapses to cause severe loss of lithiophilic interface once pressed below 40–60 kPa, which makes it unfeasible for strengthening Li metal anode at all.

**Figure 3:**
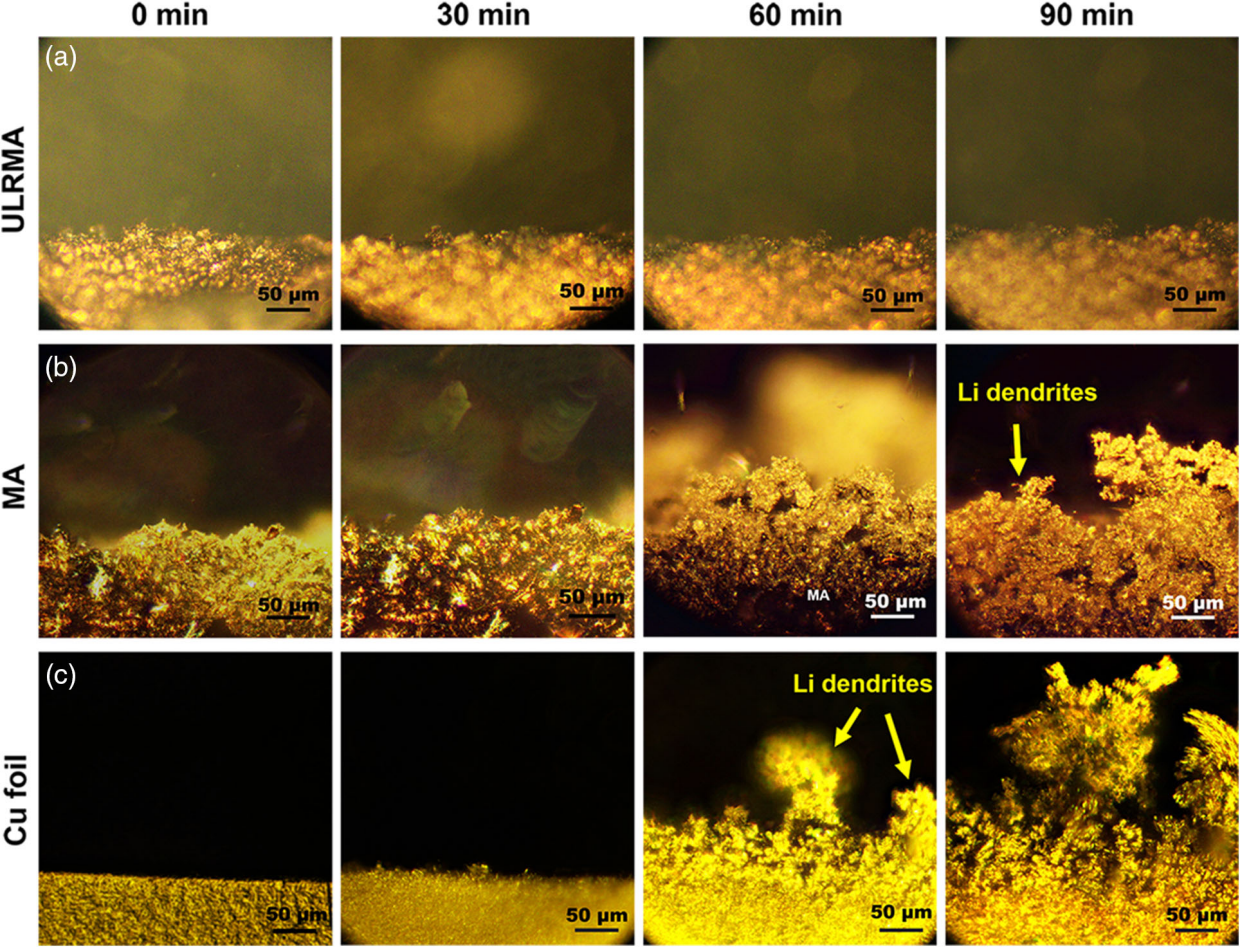
In operando optical images showing the evolution of electrode interface for different times of Li deposition on a) ULRMA, b) MA, and c) Cu foil at a current density of 1.0 mA cm^−2^.

**Figure 4:**
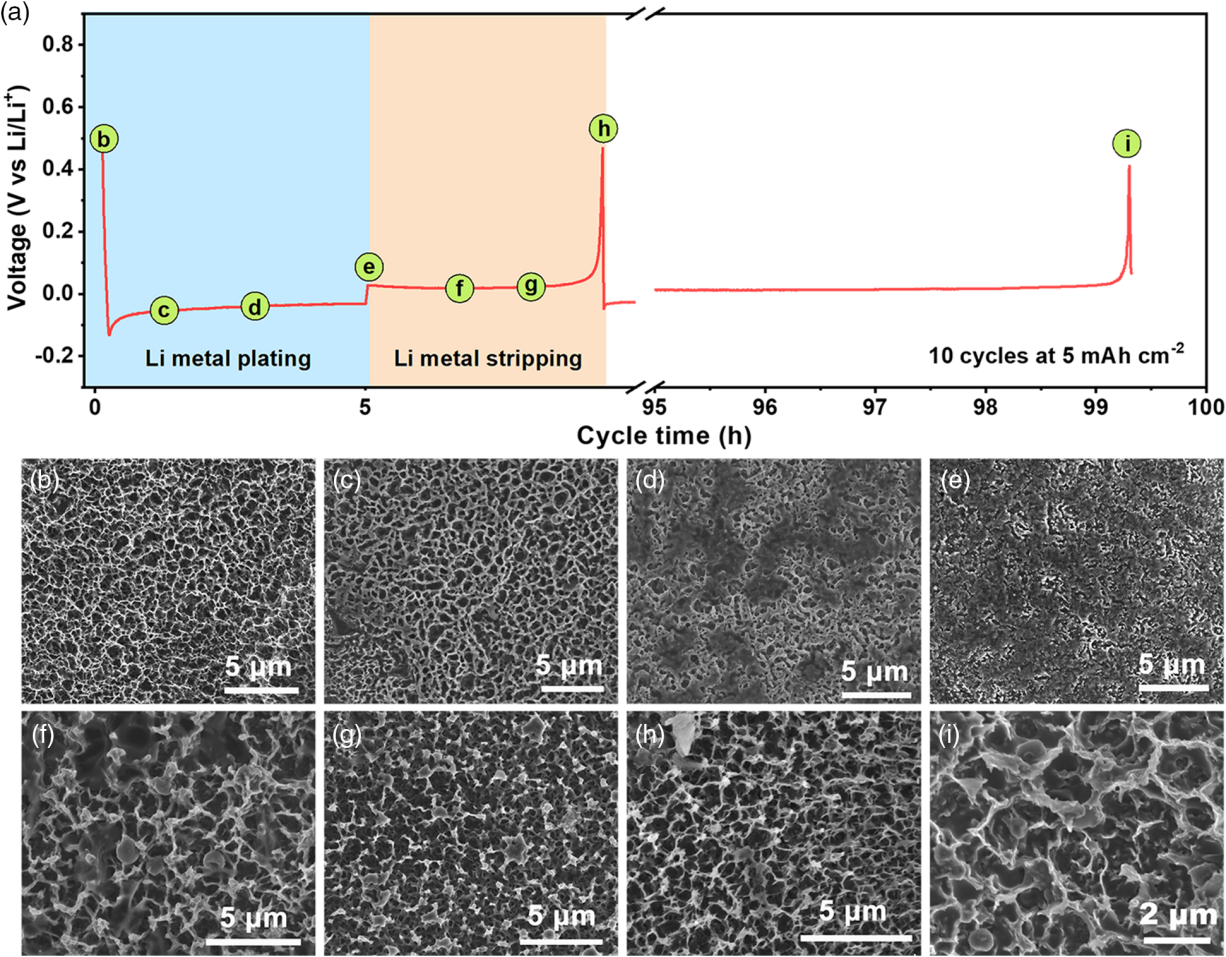
a) Galvanostatic discharge/charge curves of ULRMA at a current density of 1.0 mA cm^−2^. Corresponding SEM top‐view of Li plating on ULRMA with a capacity of b) 0.0, c) 1.0, d) 3.0, and e) 5.0 mAh cm^−2^, followed by Li stripping back to f) 4.0 mAh cm^−2^, g) 2.0 mAh cm^−2^, and h) initial state at a current density of 1.0 mA cm^−2^. i) Top‐view SEM image of ULRMA after Li stripping after ten cycles with 5.0 mAh cm^−2^ at 1.0 mA cm^−2^.

High efficiency of ULRMA for uptaking Li metal is validated by in operando optical microscopy during Li plating/stripping.^[^ ^25^ ^]^ A half‐cell with a quartz window is assembled using the ULRMA and Li foil as the working and counter/reference electrode, respectively (ULRMA||Li). The cells using MA and Cu foil as the working electrode against Li foil (MA||Li or Cu||Li) are also tested under identical conditions. The working electrodes in all the cells have a smooth surface initially. The surface morphology of ULRMA keeps smooth throughout Li deposition in 90 min at a current density of 1.0 mA cm^−2^ (**Figure** **3**a). A negligible change in electrode thickness is observed, indicating good capability for accommodating the Li metal. As a sharp contrast, Li dendrite growth on limited lithiophilic interface of MA and Cu foil is initiated in 30 min, followed by mossy growth on the electrode surface within 60–90 min (Figure 3b,c). In this process, the thickness of both electrodes increased remarkably to induce a highly coarse interface. During Li stripping, the dissolution of some Li dendrites at the root generates the “dead Li” that is electrically isolated from the electrochemical system (Figure S7a,b, Supporting Information). This phenomenon inevitably induces the loss of active Li metal and, thus, deteriorates the cell reversibility. The morphological evolution of ULRMA with Li plating/stripping going is further examined by scanning electron microscopy (SEM) analysis (**Figure** **4**a). The lithiophilic ULRMA with a highly porous structure show high efficiency for uptaking the Li metal with a capacity of 5.0 mAh cm^−2^ (Figure 4b–d). At the end of Li deposition, the ULRMA are largely filled by Li metal to form a relatively even surface free of Li dendrites (Figure 4e). This observation is constant with the Li dendrite‐free deposition on ULRMA by an in operando optical microscopy analysis (Figure 3a). Upon the next charge, the Li metal is stripped from ULRMA to gradually leave original macroporous frameworks (Figure 4f,g). After fully Li stripping at a voltage of 0.5 V, the ULRMA well retain the original texture without collapsing both the 3D architecture and macroporous frameworks because of high mechanical strength and resilience (Figure 4h). After ten cycles of Li plating/stripping, it still maintains high robustness and efficiency for uptaking Li metal at a capacity of 5.0 mAh cm^−2^ without the formation of Li dendrites or “dead Li” (Figure 4i).

**Figure 5:**
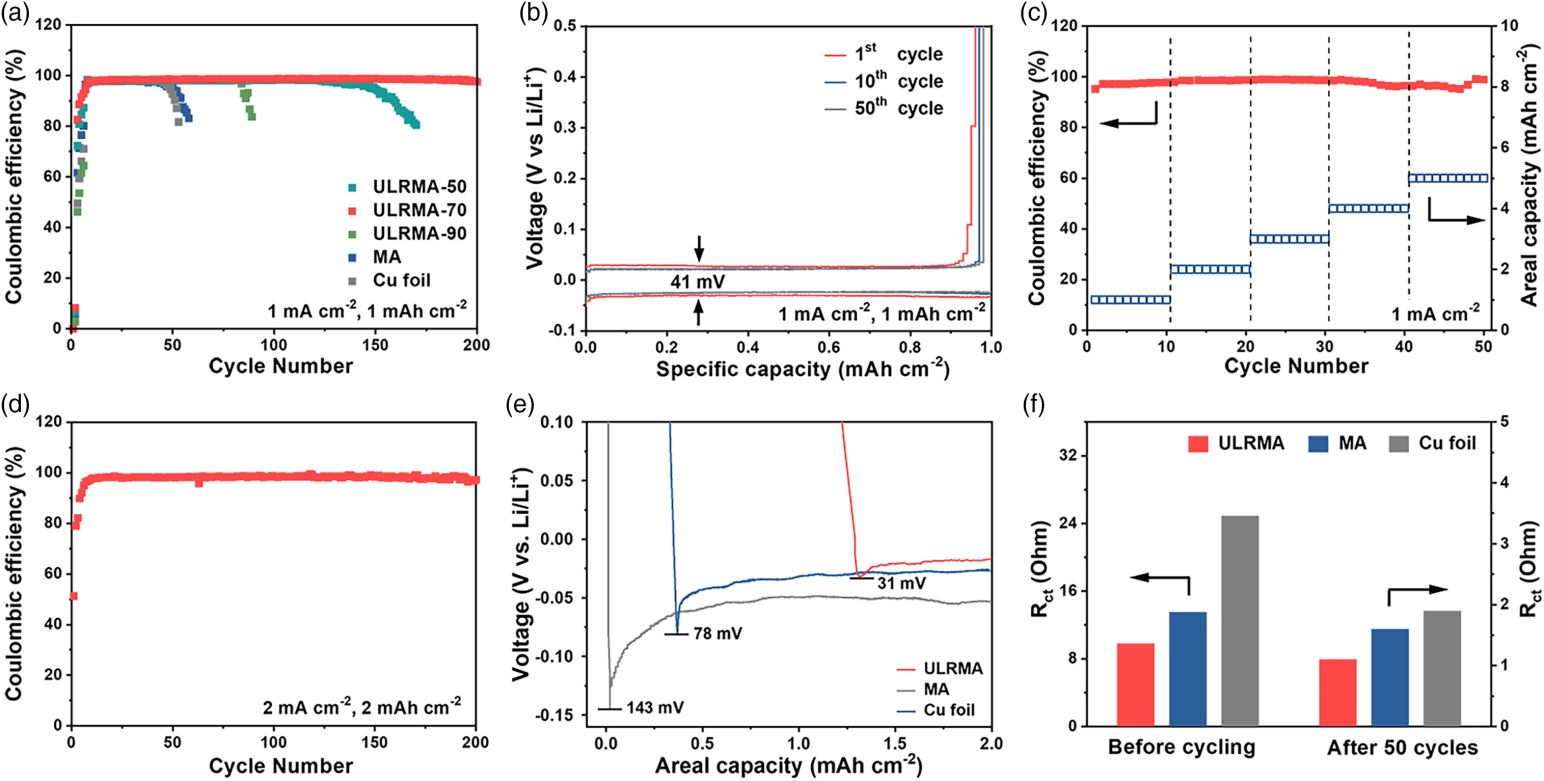
a) CE of ULRMA with 50% (ULRMA‐50), 70% (ULRMA‐70), and 90% (ULRMA‐90) MXene with a Li deposition capacity of 1.0 mAh cm^−2^ at a current density of 1.0 mA cm^−2^. The CEs of MA and Cu foil are also compared under identical conditions. b) Voltage profiles of ULRMA‐70 with a deposition capacity of 1.0 mAh cm^−2^ at 1.0 mA cm^−2^. c) CE of ULRMA‐70 with Li deposition capacities increasing from 1.0 to 5.0 mAh cm^−2^ at 1.0 mA cm^−2^. d) CE of Li plating/stripping on ULRMA‐70 for 200 cycles with a capacity of 2.0 mAh cm^−2^ at 2.0 mA cm^−2^. e) A comparison of ULRMA‐70, MA, and Cu foil in Li deposition overpotential at 1.0 mA cm^−2^. f) A comparison of ULRMA‐70, MA, and Cu foil in interfacial charge‐transfer resistance before and after Li plating/stripping for 50 cycles.

The positive effect of ULRMA on strengthening Li metal anode is further validated by the coulombic efficiency (CE) of ULRMA||Li cells in the ether‐based electrolyte. Among ULRMA with various MXene contents, the one with 70 wt% MXene exhibits the best CE of 98.8% at a current density of 1.0 mA cm^−2^ for 200 cycles (**Figure** **5**a). The gradual rise of CE at the early cycles is due to the unstable SEI film at this stage.^[^ ^26^ ^]^ The ULRMA with lower MXene content (e.g., 50 wt%) suffer from a shorter lifetime of 120 cycles due to the reduction in lithiophilic sites and conductivity, whereas the ULRMA with 90 wt% MXene exhibit an even faster CE decay as a result of reduced mechanical robustness without efficiently locking by PVA molecular chains. The PVA‐free MA shows a similar lifetime with Cu foil, although it is much lithiophilic than Cu, reflecting the significance of the high robustness of host materials in enhancing the performance of Li metal anodes. Smooth Li plating/stripping on ULRMA is indicated by a flat and long plateau in the voltage profiles with lower electrode polarization than that of MA and Cu electrodes (Figure 5b, and Figure S8a,b, Supporting Information). When the areal capacity is increased to 2.0–5.0 mAh cm^−2^, the CE of ULRMA can still maintain to 96.8–98.5%, showing high utilization and reversibility of Li metal (Figure 5c). When cycled at a current density of 2.0 mA cm^−2^ with an area capacity of 2.0 mAh cm^−2^, the CE of ULRMA could be well maintained at 97.2% for 200 cycles (Figure 5d).

With substantial lithiophilic groups on the surface, the ULRMA also work effectively in regulating the behavior of Li nucleation. The Li plating on ULRMA is initiated at around 1.8 V (vs Li/Li^+^) by Li^+^ intercalation into interlayer space of MXene and forming Li compounds with surficial groups of MXene at a current density of 1.0 mA cm^−2^ (Figure 5e, and Figure S9, Supporting Information). Such interactions dramatically reduce the overpotential to 31 mV for Li nucleation on ULRMA. In contrast, the brittle MA could not even withstand the mechanical pressure during cell assembly. The severe collapse of porous architecture and restacking of MXene largely limits the exposure of lithiophilic sites even before electrochemical cycling, resulting in a much higher Li nucleation overpotential of 78 mV. On lithiophobic and flat Cu foil, the Li nucleation has to conquer a huge overpotential of 143 mV. In this case, the Li metal prefers to nucleate at the defect area on Cu surface to trigger the uneven nucleation of Li dendrites.^[^ ^27^ ^]^ After the initial nucleation, the Li metal is deposited on ULRMA with a flat voltage plateau controlled by significantly reduced mass‐transfer overpotential (18 mV), whereas the Li deposition on MA and Cu foil needs to overcome a much higher mass‐transfer overpotential of 27 and 51 mV, respectively. The improvement of porous ULRMA electrode in interfacial compatibility leads to a comparable charge‐transfer resistance (*R*_ct_ = 9.8 Ω) with MA (13.5 Ω), which is far lower than that of flat Cu foil electrode with the inactive surface (24.9 Ω) before cycling (Figure 5f, and Figure S10a, Supporting Information). After Li plating for 50 cycles, the ULRMA still exhibit a superior *R*_ct_ (1.1 Ω) to MA (1.6 Ω) and Cu foil (1.9 Ω), which is a good indicator of uniform distribution of Li metal with stable SEI (Figure 5f, and Figure S10b, Supporting Information). Apparently, the enhancement of Li plating/stripping behavior on ULRMA is a result of the synergy of lithiophilic properties of MXene and the robust 3D porous architecture with a largely extended lithiophilic interface.

**Figure 6:**
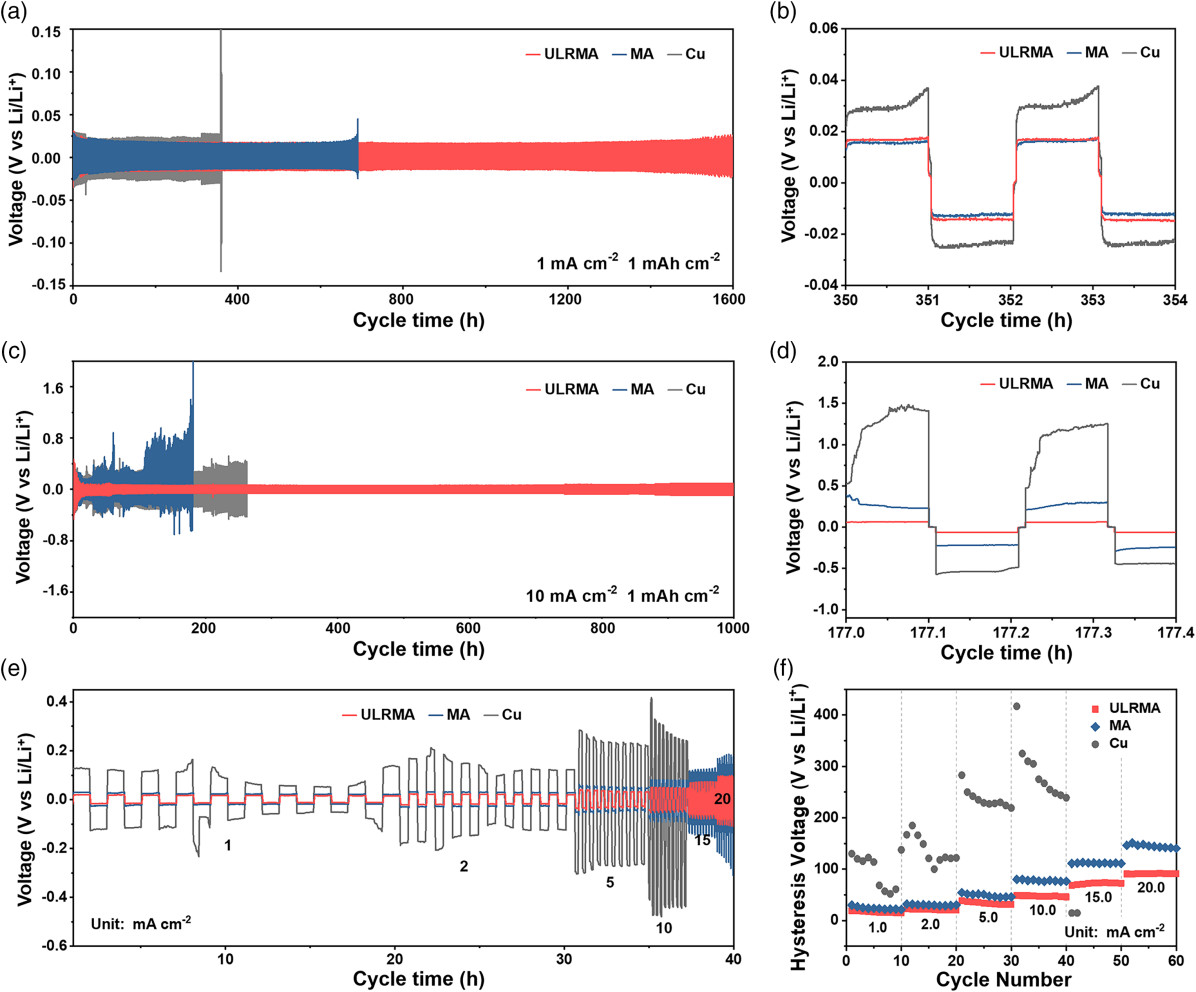
Voltage–time profiles of ULRMA, MA, and Cu foil for Li plating/stripping with a cycling capacity of 1.0 mAh cm^−2^ at a current density of a,b) 1.0 and c,d) 10.0 mA cm^−2^. e) Rate capability and f) hysteresis voltage of ULRMA, MA, and Cu foil at various current densities of 1.0–20.0 mA cm^−2^ with a cycling capacity of 1.0 mAh cm^−2^.

Long‐term voltage profiles of symmetric cells with ULRMA as the working electrode are measured at various current rates with a cycling capacity of 1.0 mAh cm^−2^. The Li plating/stripping on ULRMA exhibits a stable voltage hysteresis of 16 mV for as long as 1600 h at a current density of 1.0 mA cm^−2^ without the short circuit (**Figure** **6**a,b). As a contrast, the symmetric cells with Cu electrodes rapidly fail after 384 h under an identical current level. Replacing the Cu foil with lithiophilic MA could prolong the cell life to 690 h, which is still far inferior to ULRMA. Even cycling at a high current density of 10 mA cm^−2^, the Li||ULRMA cell can still work steadily for over 1000 h with a small voltage hysteresis of 62 mV (Figure 6c), whereas the Li||MA and Li||Cu cells show remarkable voltage fluctuation with huge voltage hysteresis (Figure 6d). Post‐mortem SEM analysis reveals that the failure of these cells can be ascribed to severe growth of Li dendrite (Figure S11, Supporting Information). This nonuniform deposition behavior would aggravate the accumulation of “dead Li” and crack of SEI layer to damage the cell performance.^[^ ^28^ ^]^ The Li||ULRMA cell also excels most of the reported Li metal anodes in terms of cycle life and current rate response due to an overall enhancement in structural stability, conductivity, and lithiophilic properties (Table S1, Supporting Information). The ULRMA with a large and conductive interface benefit in reducing local current density and the flood of Li^+^ flux upon fast Li plating/stripping at high current rates. The Li||ULRMA cells display a stable yet gradually increased overpotential of 11, 21, 32, 48, 80, and 102 mV at the current densities of 1, 2, 5, 10, 15, and 20 mA cm^−2^, respectively (Figure 6e). The low overpotentials and smooth cycling plateaus indicate the fast kinetics of Li^+^ migration throughout the robust architecture of ULRMA against high‐rate cycling (Figure 6f), while the Li||MA cell experiences a dramatic increase in overpotential at the high current densities over 10–20 mA cm^−2^. For Li||Cu cell, it exhibits huge overpotentials and a dramatic increase in voltage hysteresis with current rate increasing due to sluggish charge/mass transport and SEI damage during rapid Li plating/stripping.^[^ ^29^ ^]^

**Figure 7:**
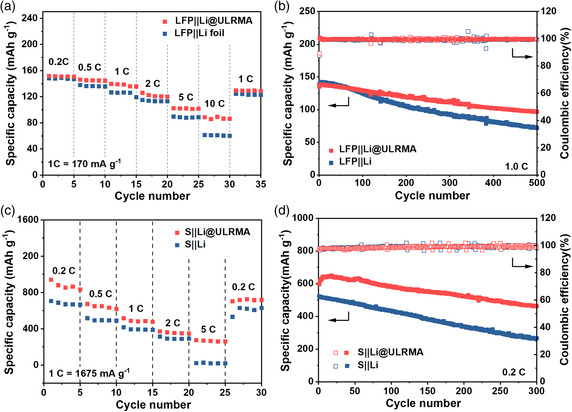
a) Rate capability of LFP||Li@ULRMA and LFP||Li full cells. b) Cycling performance and corresponding CE of both cells at 1.0 C (1 C = 170 mA g^−1^). c) Rate capability of S||Li@ULRMA and S||Li full cells. d) Cycling performance and corresponding CE of both cells at 0.2 C (1 C = 1675 mA g^−1^). Commercially available LFP and sulfur are directly used without pretreatment for all the tests.

Practical potential of Li@ULRMA anode is demonstrated in full cells by pairing with commercially available LiFePO_4_ (LFP) without nanostructuring as the cathode (LFP||Li@ULRMA). Such cells deliver superior rate performance to full cells against Li foil (LFP||Li) at high current rates above 5–10 C (1 C = 170 mA g^−1^) (**Figure** **7**a, and Figure S12, Supporting Information). During long‐term cycling, a high capacity retention of 70.4% can be achieved after 500 cycles at 1.0 C (Figure 7b), whereas the capacity of LFP||Li cells rapidly declines by 52.4% after 500 cycles under identical conditions due to poor reversibility of Li metal anode. Using Li@ULRMA anode can also significantly improve the Li–S cell performance when coupling with commercial sulfur without nanostructuring (S||Li@ULRMA). Such cells exhibit superior capacities of 270–880 mAh g^−1^ at 0.2–5 C (1 C = 1675 mA g^−1^) to the ones with Li foil as the anode. The latter lost almost all the capacities at a high current rate of 5 C (Figure 7c). After 300 cycles, the S||Li@ULRMA cells still maintain a high capacity of 450 mAh g^−1^ with a capacity retention of 77.6% and nearly 100% CE at 0.2 C. In contrast, the Li–S batteries with Li foil as the anode suffer from much lower capacities and fast capacity decay to as low as 250 mAh^−1^ after 300 cycles due to poor utilization and reversibility of Li metal anode (Figure 7d).

## 3 Conclusion

In summary, we reported a facile hydrogen‐bonding crosslinking strategy for fast assembly of MXene to ultralight aerogels with high mechanical robustness for strengthening Li metal anode. This MA well integrates molecular‐level lithiophilic sites onto hierarchically porous 3D architecture with high conductivity and build‐in stain‐resistant properties. They are capable of uptaking Li metal at a capacity up to 5.0 mAh cm^−2^ without dendrite hazards and collapse against repeated Li plating/stripping for 1600 h with a fast rate response up to 20 mA cm^−2^ and high CE. Benefited from good stability and reversibility, the Li metal anode with robust MA backbone significantly improves the capacity retention of Li‐ion and Li–S cells for over 300–500 cycles at the high current rates of 5–10 C when paired with commercially available LiFePO_4_ and sulfur cathode, respectively.

## 4 Experimental Section

4.1

4.1.1

##### Synthesis of Ti_3_C_2_T_x_ MXene

The Ti_3_C_2_T_*x*_ MXene was obtained by etching Ti_3_AlC_2_ MAX phase with LiF/HCl according to literature reports and dispersed in deionized (DI) water to form a stable colloid (10 mg mL^−1^).^[^ ^30^ ^]^

##### Synthesis of ULRMA

Typically, a solution was made by dissolving 60 mg of PVA (Mw = 146 000–186 000, Sigma‐Aldrich) in 6 mL of DI water. The ULRMA with 70 wt% MXene PVA were obtained by mixing such PVA solution with MXene colloid (14 mL) followed by ultrasonic for 60 min and freeze‐drying. The ULRMA with various MXene ratios (90 and 50 wt%) were also synthesized by changing the volume of MXene colloid used. Pure MA was fabricated via a similar way in the absence of PVA.

##### Material Characterization

The morphology of the samples was characterized with field‐emission SEM (FESEM; FEI NanoSEM 450) and transmission electron microscopy (TEM; FEI TF30). The microstructure and texture of the samples were analyzed by type‐D/Max‐III X‐ray spectrometer (XRD, Cu Kα, *λ* = 1.5406 Å) and Raman spectroscopy (Thermal Scientific DXR with an excitation wavelength of 532 nm). The surface characteristics of the samples were investigated using Thermo ESCALAB MK II XPS. The textural properties of the samples were measured by Micrometrics ASAP 2020 surface area and porosity analyzer at 77 K. The FT‐IR spectra were measured by a Nicolet‐20DXB Fourier transform IR spectrometer. In operando optical microscopy of Li deposition was conducted by metallographic microscopy (NMM‐800RF, China) using homemade cells. The mechanical properties of ULRMA were performed using rheometer (TA AR2000ex, USA) and compression tester (Instron 5567).

##### Electrochemical Measurements

The ULRMA were cut into thin circular plates with a diameter of 14 mm, a weight of about 3.0 mg, and a thickness of ≈2.0 mm as the working electrode. The CR2016 coin cells were assembled with Li foil (Zhongneng Lithium Co., AR, China) as the counter and reference electrode and 40 μL of 1.0 M lithium bis(trifluoromethylsulphonyl)imide (LiTFSI) in 1,3‐dioxolane (DOL)/1, 2‐dimethoxyethane (DME) (1:1 by volume) with 2.0 wt% LiNO_3_ additive as the electrolyte (DoDo Chem, China). The Li@ULRMA anode was prepared by pre‐plating 5 mAh cm^−2^ of Li metal onto ULRMA at a current density of 1.0 mA cm^−2^. For full‐cell tests, the LiFePO_4_‐based cathode was made by mixing commercial LiFePO_4_ (Kejing Mater. Tech. Co., Ltd., China), Super P, and polyvinylidene fluoride (PVDF; Sigma‐Aldrich) in a mass ratio of 8:1:1 with the *N*‐methyl‐2.0‐pyrrolidone (NMP) as the solvent. The areal mass loading of this cathode is ≈1.87 mg cm^−2^. The Li‐ion cells were assembled with Li@ULRMA as the anode against LiFePO_4_‐based cathode in1.0 m LiTFSI in DOL/DME (1:1 by volume) with 2.0 wt% LiNO_3_ additive. The galvanostatic charge/discharge tests were performed using a LAND CT2001A battery tester at different current densities within a cutoff voltage window of 2.2–4.1 V. For making the S cathode, an S/C composite was first made by mixing commercial sulfur (Aladdin Co., Ltd) with Super P in a mass ratio of 7:3, which was sealed in a glass container and stored at 155 °C for 12 h. The sulfur cathode was prepared by mixing the S/C composite, Super P, and PVDF in a weight ratio of 8:1:1 with NMP as the solvent. The areal mass loading of sulfur in the electrode was about 1.57 mg cm^−2^. The Li–S cells were assembled with Li@ULRMA as the anode against sulfur cathode in 1.0 m LiTFSI in DOL/DME (1:1 by volume) with 2.0 wt% LiNO_3_ additive. The galvanostatic charge/discharge tests were performed using the same battery tester between 1.7 and 2.8 V. The electrochemical impedance spectroscopy (EIS) tests were conducted by applying an alternating current (AC) amplitude of 5 mV over the frequency range from 0.01 to 105 Hz.

## Conflict of Interest

The authors declare no conflict of interest.

## Data Availability Statement

Research data are not shared.

## Supporting information

Supplementary Material
