# Supplementary material for: Hydrogen‐Bonding Crosslinking MXene to Highly Robust and Ultralight Aerogels for Strengthening Lithium Metal Anode
Source: Small Sci. 2021 Jul 16;1(9):2100021. doi: 10.1002/smsc.202100021 (PMC11936033; doi:10.1002/smsc.202100021)
Supplement: Supplementary file 1 — Supplementary Material [file SMSC-1-2100021-s001.pdf]

Supporting Information

Hydrogen-Bonding Crosslinking MXene to Highly Robust and Ultralight Aerogels for Strengthening Lithium Metal Anode

Xiangyu Meng, Yufeng Sun, Mengzhou Yu, Zhiyu Wang and Jieshan Qiu**

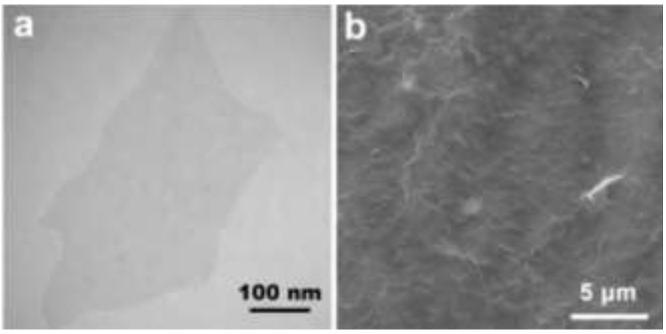

Figure S1. (a) TEM and (b) SEM image of $Ti_3C_2T_x$ MXene.

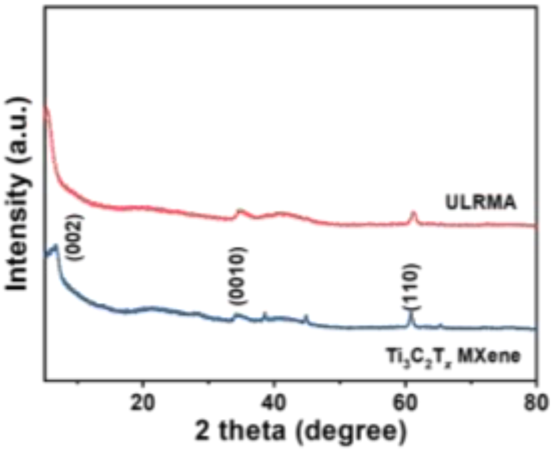

Figure S2. XRD patterns of ULRMA and pristine $Ti_3C_2T_x$ MXene.

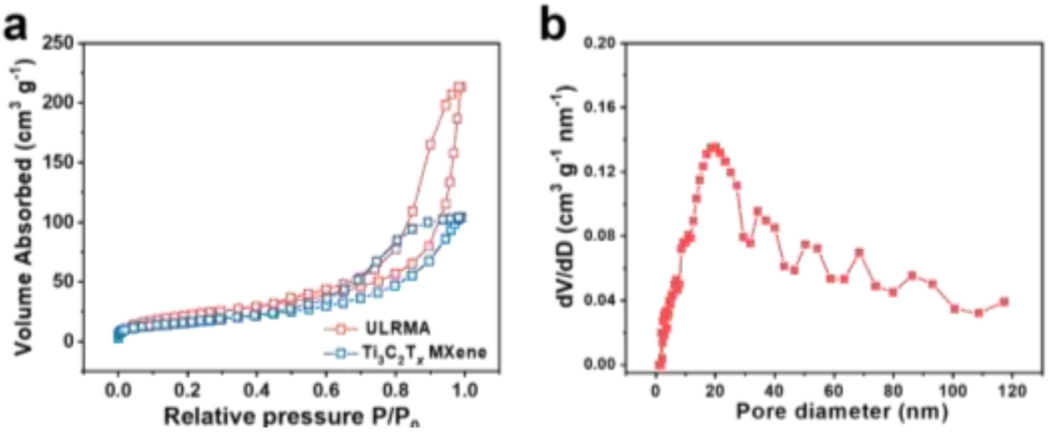

Figure S3.(a) N_2 absorption-desorption isotherms and (b) pore size distribution of ULRMA.

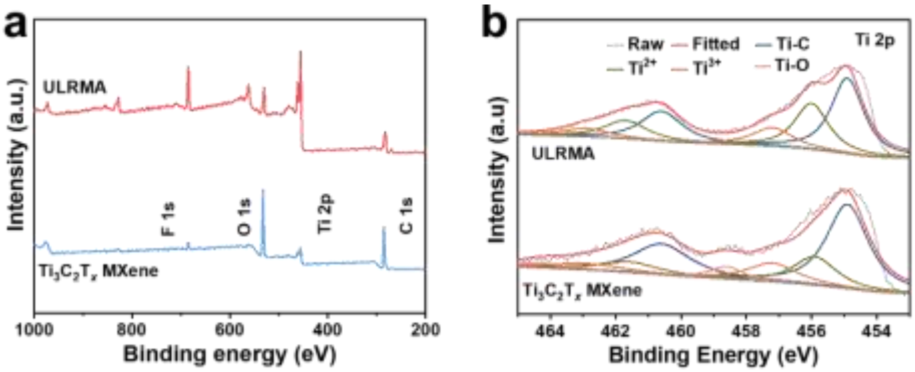

Figure S4. (a) XPS survey spectrum of ULRMA and $Ti_3C_2T_x$ MXene; (b) Ti 2p XPS spectra of ULRMA and $Ti_3C_2T_x$ MXene.

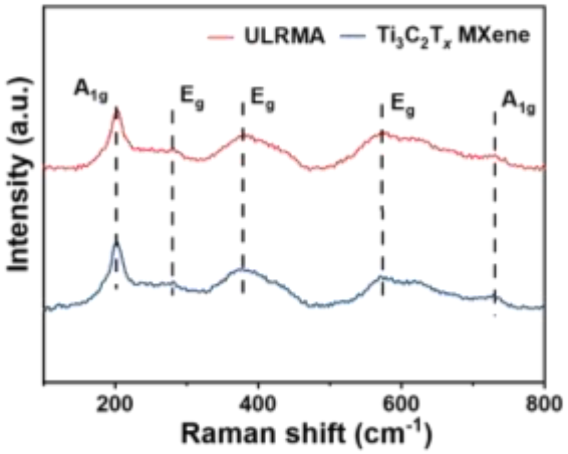

Figure S5. Raman spectra of ULRMA and $Ti_3C_2T_x$ MXene.

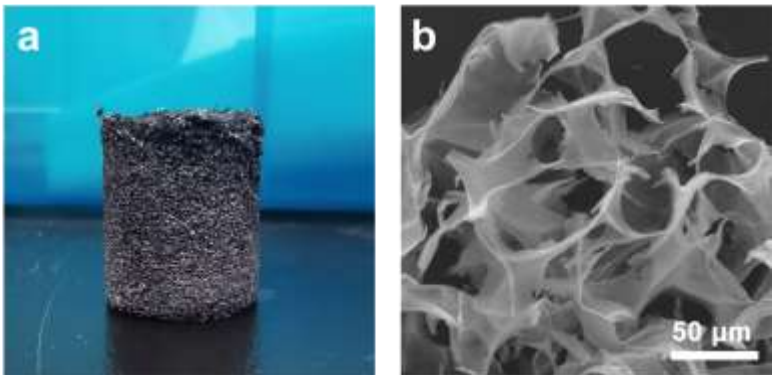

Figure S6. (a) Optical and (b) SEM image of MXene aerogel (MA).

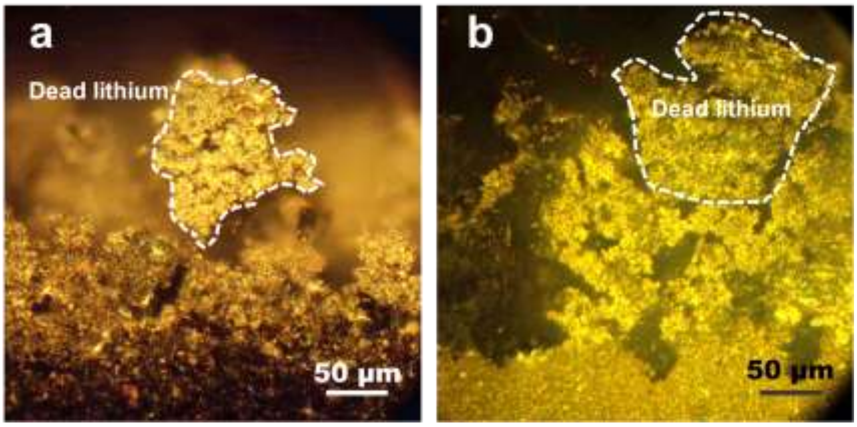

Figure S7. The formation of dead Li monitored by *in-operando* optical microscopy during Li striping from (a) MA and (b) Cu foil at a current density of 1.0 mA cm^{-2}.

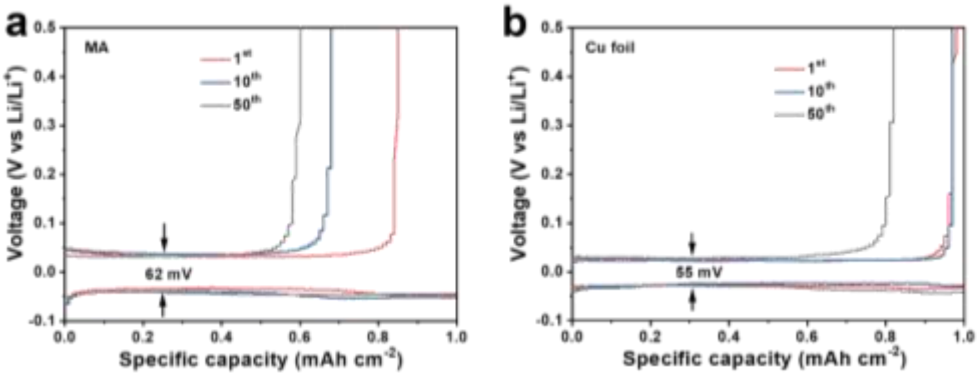

Figure S8. Voltage profiles of (a) MA and (b) Cu foil with a deposition capacity of 1.0 mAh cm^{-2} at a current density of 1.0 mA cm^{-2}.

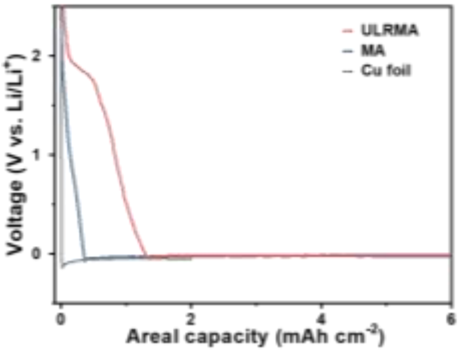

Figure S9. Galvanostatic discharge curves for Li nucleation on ULRMA, MA and Cu foil at a current density of 1.0 mA cm^{-2}.

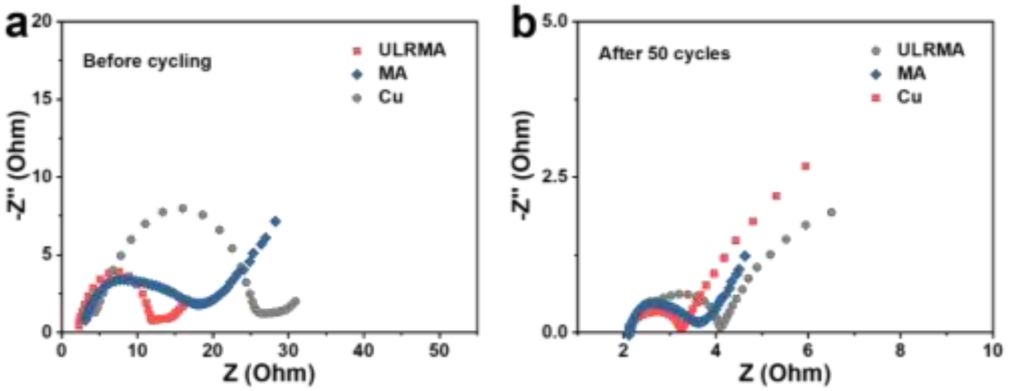

Figure S10. EIS spectra of ULRMA, MA and Cu foil (a) before and (b) after Li plating/stripping for 50 cycles over 0.01 to 10^5 Hz.

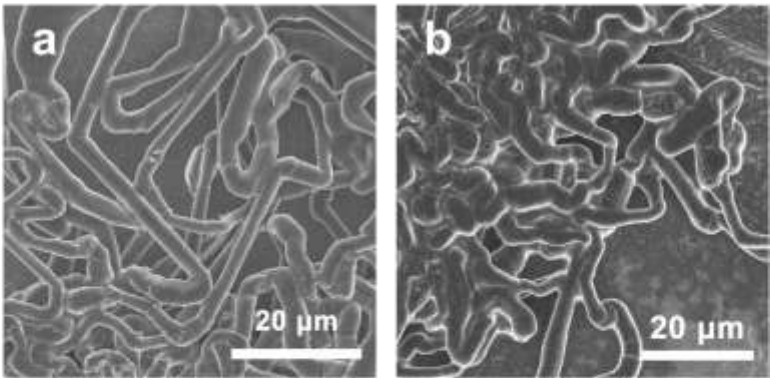

Figure S11. SEM images showing the massy growth of Li dendrites on (a) Cu foil and (b) MA after repeated Li plating/stripping with a cycling capacity of 1.0 mAh cm^{-2} at 1.0 mA cm^{-2}.

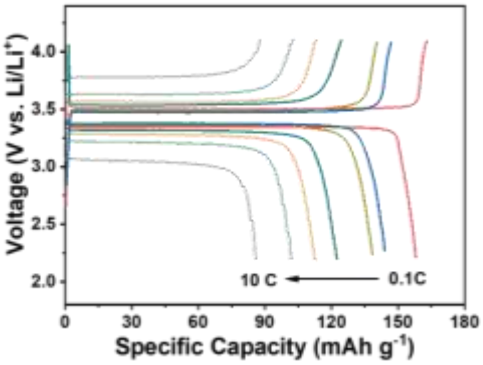

Figure S12. Galvanostatic charge/discharge curves of LFP||Li@ULRMA cell at different current rates.

Table S1. A companion between ULRMA and reported Li host in the performance for stabilizing Li metal anode.

Li metal anode	Current density / mA cm^{-2}	Cycle time / h	Reference
**ULRMA**	**1.0**<br>**10.0**	**1600**<br>**700**	**This work**
CuBr/Br-doped graphene film/Cu foam	1.0	850	*J. Am. Chem. Soc.* 2018, 140, 18051
Crumpled graphene ball	0.5	750	*Joule* 2018, 2, 184
Carbonized eggplant	10.0<br>1.0	100<br>250	*Adv. Energy Mater.* 2018, 1802720
3D Cu	0.2	600	*Nat. Commun.* 2015, 6, 8058
g-C_3N_4@Ni foam	1.0<br>2.0	900<br>500	*Adv. Energy Mater.* 2019, 1803186
3D porous Cu	0.5<br>1.0<br>2.0	1280<br>600<br>120	*Adv. Funct. Mater.* 2017, 27, 1606422
MXene/rGO aerogel	10.0	70	*Angew. Chem. Int. Ed.* 2018, 57, 1
Ti_3C_2 MXene-Li films	1.0	400	*Nano Energy* 2017, 39, 654
